# Providence Insane Asylum

**Published:** 1863-09

**Authors:** 


### PROVIDENCE INSANE ASYLUM.

We have before called the attention of our readers to the location of the Providence Insane Asylum and to the facilities here enjoyed for the care and successful treatment of this class of patients; but with its growing importance to the community, and its increasing facilities for usefulness, together with its increased number of inmates, it may be not altogether uninteresting for us again to speak somewhat in detail, of the present condition and prospective importance of this institution.

It is now a little over two years since it was consecrated to the care and treatment of the Insane, and placed under the direction of the Sisters of Charity. Sister Rosaline Brown has the general supervision, and in a great degree to her energy and ability both in its construction and management are we indebted for a most humane, well conducted, and well arranged institution for the care of this unfortunate class of invalids. We say indebted, since the blessing is inestimable to those who cannot be properly cared for at home, and are obliged to seek the protection of such an asylum; not more to them, however, than to the friends by whom they are surrounded.

In Buffalo and vicinity it has been nesessary heretofore to send those who required the restraints and moral influences of an asylum, to a distance, thus necessitating greatly increased expense and at the same time in many cases a great outlay of kind feeling, the separation being so wide and complete as to be exceedingly painful. It is not even now universally known that we have in our own city one of the most desirable resorts of the kind to be found in this country, and occasionally a patient is sent to the older and more over-crowded asylums of the interior or eastern portions of the State, more from ignorance of what may be found at home than for any love of the "far sought or dearly bought;" but of this there is no ground of complaint, since many more who have previously been inmates of other institutions, now return and choose this, in preference to all others.

It is located in one of the most healthy and attractive portions of the city, in an enclosure containing thirty acres of land finely cultivated and beautifully laid out in gardens and walks, with shade trees, groves, &c., &c., thus adding greatly to the beauty and attractions of the place and at the same time affording opportunity for employment for those who are able to enjoy it; an item of the greatest importance in the treatment of the diseases of the mind. The building contains forty-two private rooms which are nicely finished for the accommodation of patients and furnished in such style as to not only answer for their comfort, but to afford the luxuries and attractions of home. These rooms connect with spacious halls and verandas, and in this way, freedom from restraint appears complete, and yet the open air is obtained, while the greatest safety is insured. The care of the patients devolves almost wholly upon Sisters of Charity, who give their personal attention to this duty; very little of this labor is delegated to hired servants. It is astonishing to see what perfect control is often obtained by them, over even the most violent. Oftentimes five or six strong men accompany a patient to the hospital, when the sister in charge manages and controls with kind words, and firm assurances, much better. The restoration of each curable case is full of encouragement, of congratulation, and of incentive to renewed effort, but the care of the incurable maniac, calls for the exercise of the most christian patience and forbearance.

The two great objects which are mainly in view, are, the cure of the greatest possible number of those who are admitted, and the tenderest care of, and kindest attention to, the comfort, happiness and well being of those who are incurable. The first object, the cure, is dependent upon a great many circumstances and conditions not under the control of such institutions. Recent cases of insanity terminate in more or less perfect recovery in the majority of cases, if early placed under proper treatment both hygienic and medicinal; while those of long standing are much less hopeful. It is observed that few instances of perfect recovery are seen when insanity has been of more than one year's duration previous to admittance. It is largely the recent and acute cases which terminate in recovery.

At the present time the medical superintendence devolves upon ourselves, and proper or otherwise, it is unbecoming us to speak of its character.—The reputation of an Insane Asylum, and of the medical superintendent are inseperable, and we hope that nothing may be neglected on our part, which would contribute to the success and prosperity, growth and usefulness of this institution. We regard it as a solemn and important trust, committed to our keeping by those who have the interests of the Asylum at heart, and have expressed in this appointment the most unbounded confidence in our ability and fidelity. It will be our aim, as well as our pride, that the record of recovery stand as high as is consistent with the nature of the diseases we have to treat, and we pledge our best endeavor for both the cure, and care, of all who may be admitted for treatment to the Providence Insane Asylum.

Sometimes we are asked if it is not denominational in character, to which we earnestly reply, no, it is not; it is for the accommodation of the insane without any distinction. It was not built by any one denomination, it is not sustained by sectarian favor, it exerts no sectarian influence. It is a private asylum for the insane, where all may resort who desire, and obtain, we believe, unsurpassed advantages for recovery.

It is pleasant to see how all prejudice is gradually disappearing from the minds of those who know anything of the plan and management here adopted; and we find our rooms and wards filled with patients, who soon become greatly attached to those who so kindly minister to their wants.

The medical treatment of the Insane will constitute a pleasant field for observation and inquiry, and though it has been largely cultivated for the last few years, still it is open for further investigation. The hygienic management of Lunatics is really one of the most important subjects which can engage the thoughts of those who are entrusted with their care. Moral influences are potent for good or evil. It is incredible what effects are often produced upon diseased minds by what may be termed moral treatment, and though it may be confessed that medicine, as such, has only an indirect application to the disease of the mind, still we are not without means of cure. Employment, diversion, amusement, social opportunity, change of scene, &c., &c., is our moral materia medica, more powerful for good, in diseases of the mind than the actual materia medica in diseases of the body. But of all this we must speak, when by experience we have grown wise.

